# Double-Layer Graphene Mesh/PEDOT:PSS Conductive-Network-Reinforced PDMS Nanocomposites for Temperature-Insensitive Strain Sensing

**DOI:** 10.3390/s26154823

**Published:** 2026-07-30

**Authors:** Lei Wang, Zhiqiang Bai, Ruijie Han, Chaoxia Wu, Shengwei Mu

**Affiliations:** 1 Henan Key Laboratory of Advanced Cable Materials and Intelligent Manufacturing, Henan Institute of Technology, Xinxiang 453003, China; zhiqiangbai@hait.edu.cn (Z.B.); rjhan@haut.edu.cn (R.H.); wuchaoxia8226@ayit.edu.cn (C.W.); shengwei@hait.edu.cn (S.M.); 2 School of Materials Science and Engineering, Henan University of Technology, Zhengzhou 450007, China; 3 School of Computer Science and Information Engineering, Anyang Institute of Technology, Anyang 455000, China

**Keywords:** graphene mesh, conductive polymer, electromechanical behavior, temperature-insensitive strain sensor

## Abstract

Conductive polymer composite (CPC)-based wearable electronics and flexible strain sensors work in different environments, which require CPCs to show stable electrical performance at a wide range of temperatures. However, the resistance of most CPCs is generally temperature-dependent. In this work, a double-layer conductive framework was fabricated by coating highly conductive poly(3,4-ethylenedioxythiophene) polystyrene sulfonate (PEDOT:PSS) on a graphene mesh. The resulting composite, PEDOT:PSS/GM/PDMS-0.75, exhibited outstanding conductivity (8.1 S/cm), a high gauge factor (~42), excellent reliability (1000 cycles) and stable sensing performance within −30~140 °C. This work highlights the importance of PEDOT:PSS in improving the conductive stability of graphene-based strain sensors at different temperatures. Moreover, applications of sensing for human joint movement in different environments open new opportunities for temperature-insensitive CPCs.

## 1. Introduction

Strain sensors manufactured from conductive polymer composites (CPCs) serve as optimal elements for deployment in wearable electronic systems and flexible strain sensors [1,2,3]. CPCs formed by a polymer substrate incorporated with electrically conductive networks, including graphene meshes (GMs) [4,5,6], graphene foams [7,8,9], and carbon nanotubes [10,11,12], represent cutting-edge strain-sensing materials.

In real-world application scenarios, strain sensors are frequently subjected to varying environmental conditions, which requires them to retain a stable resistance response within specific temperature intervals [13]. Nevertheless, the electrical resistance of conductive composite materials is commonly susceptible to temperature fluctuations, which can be ascribed to the high thermal expansion coefficient of polymer substrates [14] and the temperature-dependent charge transport properties of conductive fillers [15]. For instance, two widely applied conductive fillers, carbon nanotubes [10] and poly(3,4-ethylenedioxythiophene) polystyrene sulfonate (PEDOT:PSS) [16,17,18], exhibit notable resistance variation as ambient temperature increases. Accordingly, building more flexible conductive networks that can accommodate the thermal expansion of polymer matrices and lowering the temperature coefficient of resistance of conductive fillers are essential for developing temperature-insensitive strain sensors [19,20,21].

In this study, a bilayer electrically conductive network was fabricated through depositing PEDOT:PSS onto the surfaces of GMs. The as-prepared composite film exhibited a high gauge factor of 40 within the 10% strain interval. Furthermore, its sensing performance remained stable across a broad temperature scope from −30 °C to 140 °C, which was ascribed to the synergistic contributions of the more ductile bilayer conductive framework and the low temperature coefficient of resistance possessed by the conductive fillers.

## 2. Materials and Methods

**Figure 1:**
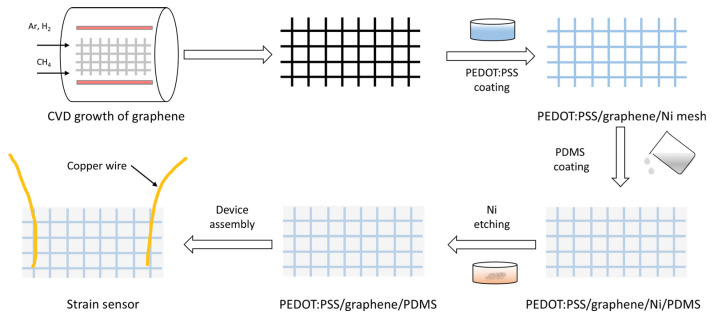
Simplified schematic preparation of composites and strain sensors.

Figure 1 illustrates the fabrication process of the double-layer conductive network composite. Commercial Ni mesh was cut into 4 cm × 1 cm strips and loaded into a CVD system (OTF-1200X-80-SL, Hefei Kejing Materials, Hefei, China). Graphene was grown on the Ni mesh surface at 1000 °C for 20 min, with Ar, H_2_ and CH_4_ flows of 500, 200 and 30 standard cubic centimeters per minute, respectively. The as-prepared graphene/Ni mesh was then immersed in a 0.75 wt% DBSA aqueous solution to improve the hydrophilicity and wettability of the graphene layers [22]. PEDOT:PSS dispersions were prepared by adding x mL of pristine PEDOT:PSS into 3 mL of deionized water, where x = 0.25, 0.5, 0.75 and 1 mL, followed by ultrasonication for 30 min. The sample was immersed in the PEDOT:PSS solution for 5 min and dried in a vacuum oven at 80 °C for 1 h. This immersion-drying process was repeated until all solution was consumed, forming a uniform double-layer PEDOT:PSS/graphene conductive network.

PDMS was diluted with ethyl acetate to prepare a 0.2 g/mL homogeneous solution. Then, it was deposited onto the PEDOT:PSS/graphene/Ni mesh surface, followed by thermal curing at 80 °C for 2 h. Subsequently, the Ni framework was removed by etching with concentrated HCl (3M) at 70 °C and then rinsed with DI water. Finally, the samples were dried in a vacuum oven at 80 °C for 24 h to thoroughly remove residual water. The composite films were denoted as PEDOT:PSS/GM/PDMS-x.

For the fabrication of strain-sensing devices, thin copper wires were firmly bonded onto both terminal sides of the as-fabricated composite conductive films using conductive silver paste. This bonding strategy was adopted to minimize interfacial contact resistance between the metal wire electrodes and the conductive composite layers, which effectively eliminates signal distortion during subsequent electrical testing [23]. The free ends of the copper wires were then connected to an external power supply for strain-sensing measurements.

The surface microstructure was characterized via scanning electron microscope (SEM) (Zeiss EVO-18, Cambridge, UK). Raman spectroscopy (HR800, Jobin Yvon, Longjumeau, France) was employed to analyze the crystalline structure of the samples. A four-point probe tester (RTS-8, Guangzhou 4 Probe Tech Co., Ltd., Guangzhou, China) was adopted to test electrical conductivity. The dynamic strain-sensing performance of the composites was recorded using an electromechanical test platform, which consists of a universal mechanical testing machine (HF-9002, Jiangsu Li Gao Testing Equipment Co., Ltd., Suzhou, China) and a digital multimeter. Copper wires were attached to sample ends with silver conductive adhesive to reduce contact resistance, and specimens were secured to tensile fixtures. The samples were cut into 15 × 3 mm strips with a 6 mm effective gauge length. Pre-stretching was applied before resistance measurement under tension to stabilize electrical signals [24]. The sensitivity of the sensor is reflected by the gauge factor (GF), which is defined as GF = ΔR/(R_0_ ε), where ΔR, R_0_ and ε are the resistance change, initial resistance, and strain of the composites, respectively [25].

The strain resistance responses of composites were tested at −30–140 °C using an electronic universal tester with a temperature chamber (CMT5105, Jinan Meters Testing Technology Co., Ltd., Jinan, China). The specimens were mounted on internal tensile fixtures, and stretching began once the preset temperature stabilized. Resistance testing during tension followed the same two-point method used at room temperature.

## 3. Results

**Figure 2:**
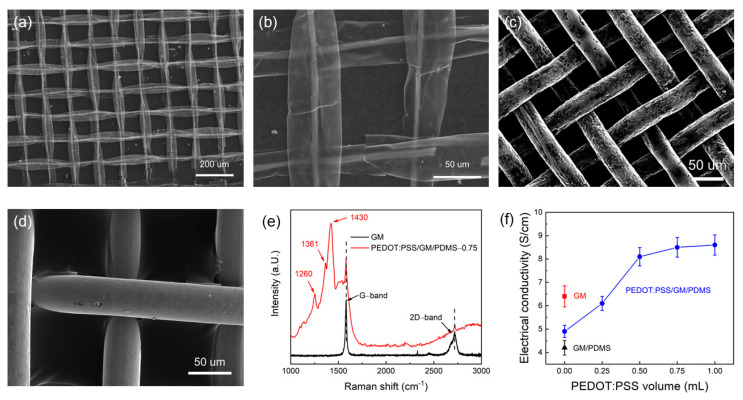
SEM images of (**a**) free-standing GMs, (**b**) free-standing GMs with higher magnification, (**c**) GM/Ni meshes, and (**d**) PEDOT:PSS/GMs/Ni-0.75; (**e**) Raman spectra and (**f**) electrical conductivities of different materials.

GMs were grown on Ni mesh templates by the CVD method under 4 vol% of CH_4_. After the etching process, the as-obtained free-standing GMs inherited the original structure of the Ni meshes, as can be seen from the SEM images in Figure 2a. The SEM image of free-standing GMs with higher magnification (Figure 2b) shows wrinkles and cracks on the film, which arise from the grain boundaries on the surfaces of the Ni meshes (Figure 2c). Figure 2e shows the Raman spectra of the GMs. It can be seen from the spectrum that the overall curve exhibits two distinct prominent peaks, namely, the G peak and the 2D peak, which are located near 1580 and 2700 cm^−1^, respectively. Notably, the D peak near 1350 cm^−1^, which is a characteristic signal of defects in graphene sheets, is absent in this spectrum. This confirms that the as-prepared graphene film possessed a perfect graphitic structure with negligible defects [26]. In addition, the 2D peak at 2700 cm^−1^ shows a broad profile with peak splitting, demonstrating that the synthesized graphene sheets adopt a multilayer structure [27].

After coating PEDOT:PSS, the resistive microcracks and grain boundaries in the GMs disappeared, as can be seen in Figure 2d. Predominant characteristic peaks at 1430, 1260 and 1361 cm^−1^ are observed in the Raman spectrum of PEDOT:PSS/GM/PDMS-0.75, corresponding to the symmetric stretching vibration of the aromatic C=C bond and the C-C and C-C stretching deformation in PEDOT:PSS, respectively [28], further suggesting the successful coating of PEDOT:PSS (Figure 2e).

The freestanding GM delivered an electrical conductivity of 6.5 S/cm, benefiting from its intrinsically defect-free graphene skeleton (Figure 2f). The electrical conductivity of GM/PDMS was reduced to 4.2 S/cm, which was caused by minor damage to the conductive graphene networks from internal stresses generated during the construction of the PDMS matrix [29]. After introducing a high electrical conductivity of approximately 850 S/cm into PEDOT:PSS [30], the electrical conductivity of the composites was significantly improved to 4.6 S/cm, with a PEDOT:PSS volume of 0.25 mL. When increasing the PEDOT:PSS volume to 0.75 mL, the electrical conductivity of PEDOT:PSS/GM/PDMS-0.75 increased to 8.4 S/cm. The highly conductive PEDOT:PSS coating can serve as efficient conductive bridges to connect these resistive microcracks and grain boundaries within GMs, which fundamentally promotes the overall electrical conductivity. Further increasing the PEDOT:PSS dosage to 1.0 mL contributed little to the conductivity, which was caused by saturated conductive pathways.

**Figure 3:**
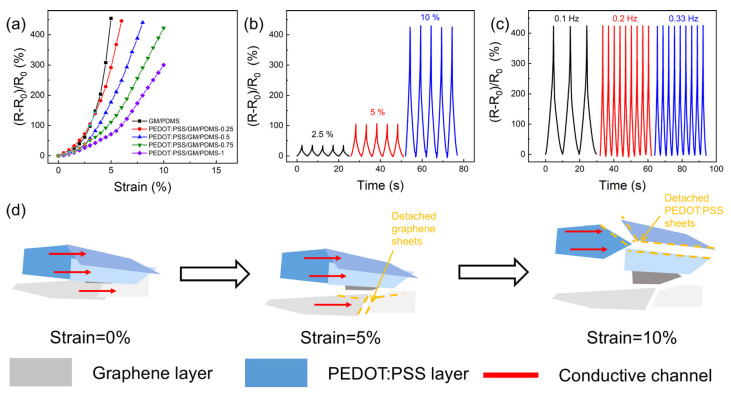
(**a**) Normalized resistance changes of composites with different PEDOT:PSS contents under stretching; resistance changes of PEDOT:PSS/GM/PDMS-0.75 during (**b**) load–release at different tensile strains and (**c**) different frequencies; and (**d**) schematic illustration of structural evolution of graphene layer and PEDOT:PSS layer in PEDOT:PSS/GM/PDMS-0.75 during stretching process.

As shown in Figure 3a, the resistance of GM/PDMS increased by ~200% at a tensile strain of 5%. Although a high GF value of 79 was obtained, the narrow sensing range restricted the actual application. The sensing performance of the composites improved with the PEDOT:PSS coatings due to the double-layer conductive network. PEDOT:PSS/GM/PDMS-0.75 exhibited increased resistance of 415% at 10% strain, showing a high average GF value of 41.5 over the entire sensing range (0~10%). With a higher coating content, the GF value of the composites slightly decreased, resulting from excessive PEDOT:PSS. Figure 3b presents the dynamic electromechanical response of PEDOT:PSS/GM/PDMS-0.75 under cyclic tensile loading–unloading tests. Under a fixed tensile strain of 10%, the sensor maintained stable resistance signals across different testing frequencies (Figure 3c).

Figure 3d shows the changes in the conductive channels during the stretching process for GM/PDMS and PEDOT:PSS/GM/PDMS-0.75. Grey sheets denote graphene layers, blue regions represent PEDOT:PSS layers, and red arrows mark continuous conductive channels. In the pristine state, the graphene and PEDOT:PSS sheets overlapped tightly to form intact, continuous conductive channels for efficient charge transfer. At 5% strain, the applied tension pulled apart the graphene sheets, breaking the conductive pathways formed by the graphene, resulting in conductivity loss in GM/PDMS with a single conductive layer. For PEDOT:PSS/GM/PDMS-0.75, the PEDOT:PSS layer dominated charge transport, endowing the composite with sustained electrical conductivity. At 10% strain, severe stretching led to massive detachment of both the graphene and PEDOT:PSS sheets, resulting in a rapid increase in resistance. Therefore, despite a minor reduction in the GF value, PEDOT:PSS/GM/PDMS-0.75 exhibited a superior sensing performance compared with GM/PDMS for a much larger strain-sensing range.

**Figure 4:**
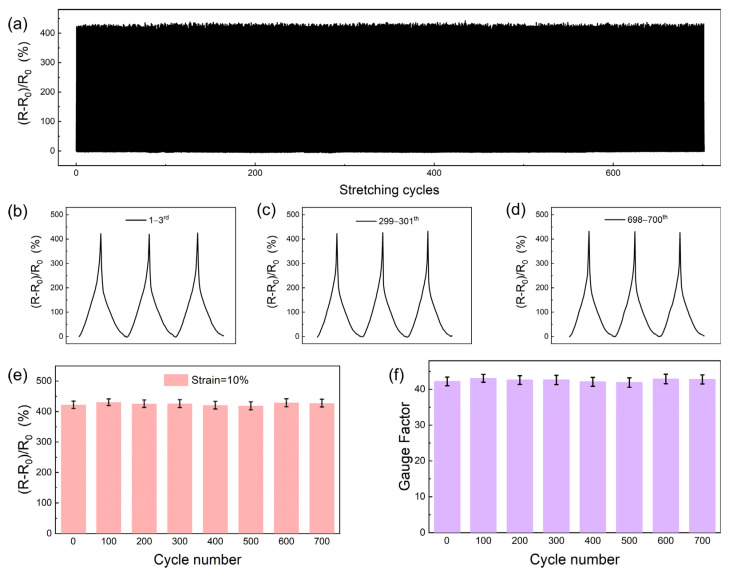
Curve of normalized resistance changes of PEDOT:PSS/GM/PDMS-0.75 over (**a**) 700 stretching–releasing cycles, (**b**) 1st–3rd cycles, (**c**) 299–301st cycles and (**d**) 698–700th cycles at a target strain of 10%. (**e**) Resistance change and (**f**) GF value at tensile strain of 10% during 700 stretching–releasing cycles.

Furthermore, long-term stretch–release cycle tests with a target strain of 10% were carried out on PEDOT:PSS/GM/PDMS-0.75 for 700 cycles, and the corresponding results are presented in Figure 4. When the composite was first stretched to 10%, its resistance increased by 422%, and this resistance state remained stable throughout the subsequent 700 cycles, as shown in Figure 4a. Figure 4b–d display the relative resistance variations in three cycle intervals: 1–3, 299–301 and 698–700 cycles. All curves show identical response profiles, with a consistent resistance peak of 420~430% at 10% strain and full baseline recovery after unloading. Figure 4e plots the relative resistance variation across cycling. The resistance signal remains nearly unchanged over the full test, with minor fluctuations, revealing reproducible electrical responses under repeated tensions. Figure 4f illustrates the corresponding GF value, which maintained a steady high value (~43) without obvious decay, indicating well-preserved sensing performance. These results confirm that the PEDOT:PSS/GM/PDMS-0.75 composite possesses outstanding cycling stability and durable sensing performance for strain detection.

**Figure 5:**
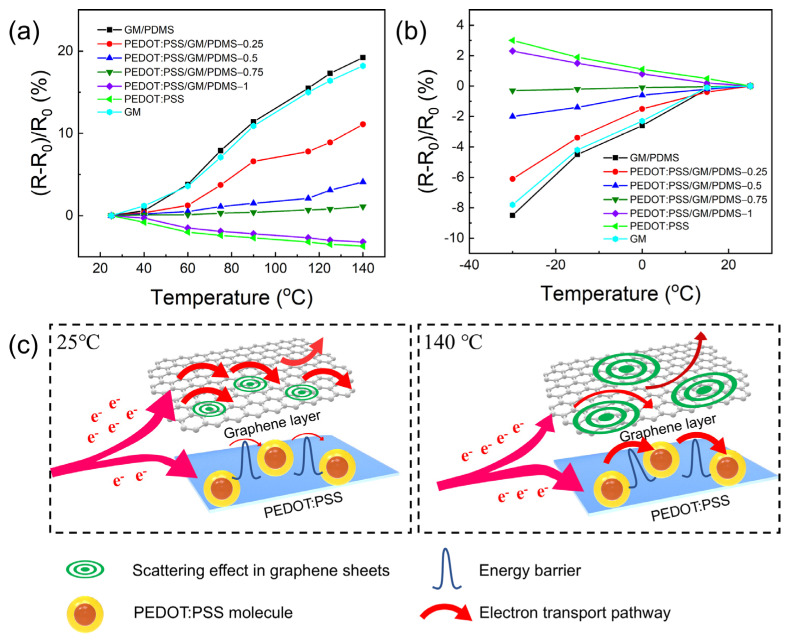
Normalized resistances of composites with different PEDOT:PSS contents in the temperature ranges of (**a**) 25–140 °C and (**b**) −30–25 °C; (**c**) schematic diagram of the electron transport in double-layer conductive networks.

It is essential for strain sensors to maintain temperature-insensitive resistance under mechanical deformation. Herein, the temperature dependence of the composite electromechanical performance was systematically investigated (Figure 5), where R_0_ refers to the resistance at 25 °C. Figure 5a presents the resistance evolution of different materials within the temperature range of 25 °C to 145 °C. When the composite only contained a monolayer conductive network composed of graphene (GM/PDMS), the overall resistance of the material increased with rising temperature. At 60 °C, the material resistance rose by 3.8%; when the temperature increased to 90 °C, the resistance increased by 11.4%; and at 140 °C, the resistance of the material was 19.2% higher than its initial value. There are two primary mechanisms responsible for this phenomenon. First, CVD-grown graphene sheets exhibited intrinsic metallic character. Elevated temperatures intensify phonon-carrier scattering on graphene surfaces, hindering electron transport across graphene sheets and, consequently, increasing the overall resistance of the composite [31]. Second, the PDMS matrix and graphene sheets possess a substantial mismatch in thermal expansion coefficients. Thermal expansion of the matrix upon heating induces microcracks in partial graphene frameworks, disrupting the continuous conductive network and raising the material resistance. The resistance–temperature variation curve of pure GM is similar to that of GM/PDMS composites, indicating that the mismatch of thermal expansion coefficients between the filler and matrix is not the dominant factor contributing to the rise in resistance.

PEDOT:PSS is a semiconducting conductive polymer with a distinctive core–shell structure, whose electrical conductivity exhibits a negative correlation with temperature. Specifically, as temperature rises, electrons on the conductive PEDOT chains are thermally excited and can more readily surmount the energy barriers of insulating PSS segments, enhancing the electron transport capacity of PEDOT:PSS and reducing its overall resistance. In contrast, at lower temperatures, electrons lack sufficient thermal energy to overcome the energy barriers of PSS molecules, leading to an increase in the total resistance of PEDOT:PSS [32]. As shown in Figure 5a, the resistance of the neat PEDOT:PSS film decreased by 3.7% at 140 °C compared with that at 25 °C.

After introducing PEDOT:PSS into the GM network to modify it, the normalized resistance variation of the composite was greatly suppressed. When the temperature increased from 25 °C to 140 °C, the normalized resistance of PEDOT:PSS/GM/PDMS-0.75 only slightly increased by 1.1%. According to Figure 5b, PEDOT:PSS/GM/PDMS-0.75 underwent a resistance decrement of 0.3% in the temperature range of 25 °C to −30 °C, indicating outstanding temperature stability. Such a compensation effect of electron transport against temperature fluctuation has been verified in other materials with analogous structures [33].

To better elucidate the outstanding resistance stability of composites with double-layer conductive networks under various temperature conditions, the electron transport processes within graphene layers and PEDOT:PSS layers at room temperature and 140 °C were schematically illustrated, as presented in Figure 5c. Electrons migrate freely on the surfaces of graphene sheets, and their transport is primarily governed by surface phonon scattering. In contrast, electrons move through PEDOT:PSS chains via hopping transport, and the quantity of excited-state electrons capable of surmounting intermolecular energy barriers dominates the electrical conductivity of PEDOT:PSS. At an elevated temperature of 140 °C, phonon scattering on the graphene sheet surfaces intensified, reducing the number of mobile electrons and thereby degrading the conductive performance of the graphene layer. Simultaneously, a larger population of electrons along the conductive PEDOT chains gained sufficient kinetic energy at high temperatures to overcome intermolecular energy barriers, which enhanced the conductivity of the PEDOT:PSS layer. The competition between the different temperature responses of graphene and PEDOT:PSS led to a reduced temperature coefficient of resistance of the conductive framework.

**Figure 6:**
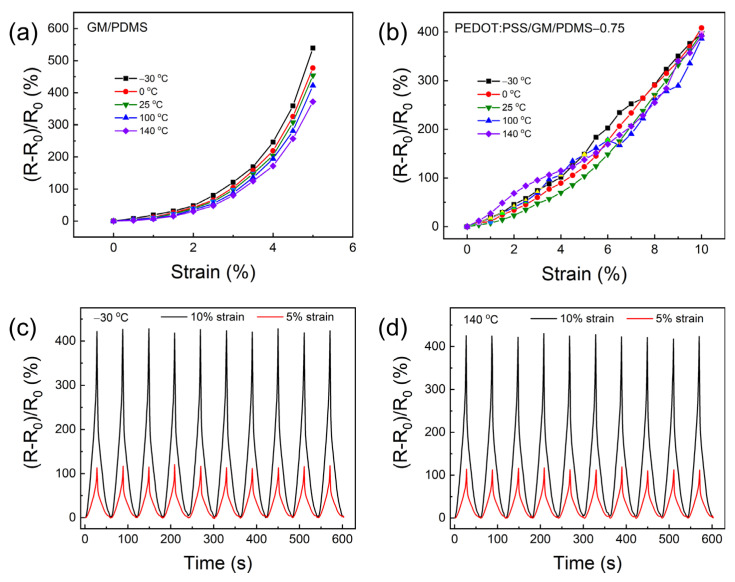
Normalized resistance of (**a**) GM/PDMS and (**b**) PEDOT:PSS/GM/PDMS-0.75 during stretching at different temperatures; cyclic stretching with maximum strains of 5% and 10% at temperatures of (**c**) −30 °C and (**d**) 140 °C for PEDOT:PSS/GM/PDMS-0.75.

The resistance of GM/PDMS and PEDOT:PSS/GM/PDMS-0.75 stretched at different temperatures was examined. For GM/PDMS, the relative resistance variation increased nonlinearly with tensile strain within a narrow measurable range of 0~5%, as shown in Figure 6a. The corresponding normalized resistance variations at 5% strain reached 539.1%, 477.1%, 454.2%, 422.4% and 372.1% at −30 °C, 0 °C, 25 °C, 100 °C and 140 °C, respectively. High temperature weakens the sensing signal and restricts its application. After introducing a PEDOT:PSS conductive layer, the PEDOT:PSS/GM/PDMS-0.75 composite exhibited greatly improved sensing performance. Its detectable strain range was extended to 0–10%, with the same temperature–response trend observed, as shown in Figure 6b. The corresponding normalized resistance variations at 10% strain reached 396.7%, 408.4%, 397.8%, 386.4% and 391.7% at −30 °C, 0 °C, 25 °C, 100 °C and 140 °C, respectively. Even at extreme temperatures of 140 °C and −30 °C, the sensor presented comparable normalized resistance profiles during stretching and releasing, with only minor numerical deviations. Dynamic cyclic tests at −30 °C (Figure 6c) and 140 °C (Figure 6d) further verified its robust wide-temperature stability. Under cyclic loading of 5% strain, the maximum resistance variation remained at around 5% at both −30 °C and 140 °C. When the tensile strain increased to 10%, the peak resistance change was approximately 10% at the two temperature extremes, which was in good agreement with the uniaxial tensile test results. Overall, the incorporation of PEDOT:PSS endowed PEDOT:PSS/GM/PDMS-0.75 with an expanded sensing range and stable strain detection capability over a broad temperature window of −30 °C to 140 °C, far outperforming GM/PDMS composites.

Table 1 compares PEDOT:PSS/GM/PDMS-0.75 with published PEDOT:PSS-based strain sensors in terms of sensing range, GF value and temperature effects. The existing works exhibit obvious performance tradeoffs: many high-GF sensors only support ultra-narrow sensing ranges, while sensors with broad working strain ranges deliver low GF values; many prior studies also omit temperature-dependent resistance characterization. In contrast, the sensor in this work achieves a high GF of 42 under 10% strain alongside outstanding thermal stability, with resistance remaining constant within the wide temperature window from −30 °C to 140 °C. Overall, this work attains a favorable balance between high strain sensitivity and broad thermal operating tolerance, outperforming most compared PEDOT:PSS-based strain sensors for practical variable-temperature sensing applications.

**Figure 7:**
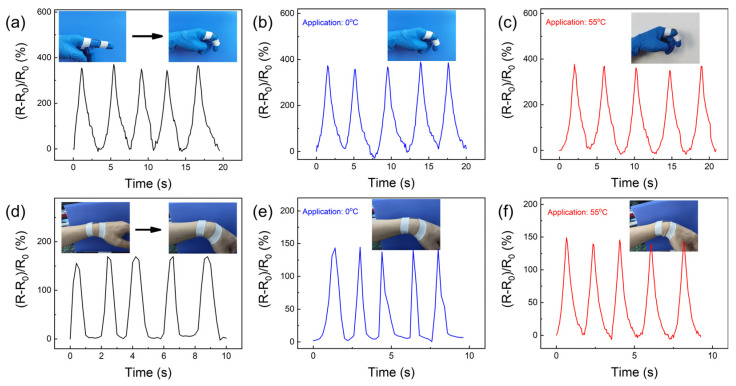
Application of PEDOT:PSS/GM/PDMS-0.75 composites: finger bending at (**a**) room temperature, (**b**) 0 °C and (**c**) 55 °C; wrist bending at (**d**) room temperature, (**e**) 0 °C and (**f**) 55 °C.

Demonstrations of PEDOT:PSS/GM/PDMS-0.75 as strain sensors for flexible and soft devices are shown in Figure 7. Changes in the strain-sensing performance under low-, room- and high-temperature conditions were investigated. The composite sensor maintained stable sensing performance at 0 °C, ambient temperature, and 55 °C, respectively. These results verify that the PEDOT:PSS/GM/PDMS-0.75 sample can satisfy the strain-monitoring demands of dynamic human movements, which endows it with superior reliability and broad applicability for all-weather wearable electronic devices.

## 4. Conclusions

In summary, a strain sensor consisting of a double-layer PEDOT:PSS/GM conductive network was fabricated. High conductivity of 8.1 S/cm was realized owing to PEDOT:PSS forming conductive interconnections to cover the microcracks and grain boundary defects with high resistance in GMs. PEDOT:PSS/GM/PDMS-0.75 exhibited a GF value of 41.5 at 10% strain and outstanding reproducibility. Furthermore, stable sensing properties were achieved under cyclic deformations over the temperature range of −30~140 °C, which significantly broadened the practical application situations.

## Figures and Tables

**Table 1:** Comparison of PEDOT:PSS/GM/PDMS-0.75 with reported works.

Ref	Materials	Sensing Range	GF Value	Temperature Effects
[34]	PEDOT:PSS/PDMS	150%	17.1	N/A
[35]	PEDOT:PSS/natural rubber	10%	123.8	N/A
[36]	PEDOT:PSS/CNT/PDMS	50%	9	N/A
[37]	Fe nanowires/graphene/PEDOT:PSS	80%	7.8	N/A
[38]	Graphene/Pd/PEDOT:PSS	60%	18	N/A
[39]	PEDOT:PSS/RGO/PDMS	21%	8.3	Resistance changes at 30~50 °C
[40]	PEDOT:PSS/graphene	50%	1.9	Resistance increases with temperature
[41]	DMSO-doped PEDOT:PSS film	2%	2	Resistance keeps stable at 20~100 °C
[19]	Graphene foam/PEDOT:PSS/PDMS	80%	0.15	Resistance keeps stable at −30~145 °C
This work	Graphene mesh/PEDOT:PSS/PDMS-0.75	10%	42	Resistance keeps stable at −30~140 °C

## Data Availability

The original contributions presented in this study are included in the article. Further inquiries can be directed to the corresponding author.
